# Impact of the CamAPS FX hybrid closed‐loop insulin delivery system on sleep traits in older adults with type 1 diabetes

**DOI:** 10.1111/dom.14914

**Published:** 2022-11-15

**Authors:** Hood Thabit, Charlotte Boughton, Womba Mubita, Jose Rubio, Julia K. Mader, Parth Narendran, Mark Evans, Lalantha Leelarathna, Malgorzata E Wilinska, Catherine Fullwood, Alice M Gregory, Roman Hovorka, Martin K Rutter

**Affiliations:** ^1^ Centre for Biological Timing, Diabetes, Endocrinology and Metabolism Centre Manchester University NHS Foundation Trust, Manchester Academic Health Science Centre Manchester UK; ^2^ Division of Diabetes, Endocrinology and Gastroenterology, Faculty of Biology, Medicine and Health University of Manchester Manchester UK; ^3^ Wellcome ‐MRC Institute of Metabolic Science, University of Cambridge Cambridge UK; ^4^ Wolfson Diabetes and Endocrine Clinic, Cambridge University Hospitals NHS Foundation Trust Cambridge UK; ^5^ Division of Endocrinology and Diabetology, Department of Internal Medicine Medical University of Graz Graz Austria; ^6^ University Hospitals Birmingham NHS Foundation Trust Birmingham UK; ^7^ Institute of Immunology and Immunotherapy, University of Birmingham Birmingham UK; ^8^ Research & Innovation, Manchester University NHS Foundation Trust Manchester UK; ^9^ Centre for Biostatistics, Division of Population Health, Health Services Research and Primary Care, Faculty of Biology, Medicine and Health University of Manchester, The Manchester Academic Health Sciences Centre Manchester UK; ^10^ Department of Psychology, Goldsmiths University of London London UK

**Keywords:** randomized trial, type 1 diabetes, insulin pump therapy, continuous glucose monitoring (CGM)

## 1 INTRODUCTION

Healthy sleep supports general health and well‐being.^1^, ^2^ Sleep disturbance in type 1 diabetes (T1DM) can be caused by factors including fear of hypoglycaemia and suboptimal glucose control.^3^ For example, hypoglycaemia or hyperglycaemia can disturb sleep, through mechanisms including sympathetic activation and neuropathic pain, respectively.^4^

Several observational studies have suggested that hybrid closed‐loop (HCL) system initiation is associated with improved sleep,^5^, ^6^ but these data could be limited by confounding and other biases. Moreover, different HCL devices could have differing effects on sleep; outcomes could be influenced by user age and the measures by which sleep is assessed.

Our objectives, in older adults with T1DM, were to: (a) assess the impact of an adaptive HCL system (CamAPS FX) on subjectively and objectively assessed sleep traits in a post hoc analysis of randomized controlled trial (RCT) data; and (b) assess the relationship between glucose control and sleep traits in the trial cohort.

## 2 METHODS

In a previously reported crossover RCT, glucose control with the CamAPS FX HCL system was compared with sensor‐augmented pump (SAP) therapy in older adults aged >60 years with T1DM.^7^ Following randomization, participants initially assigned to the CamAPS FX HCL device were appropriately trained and then used this system for 16 weeks. During the SAP period, participants used the same devices but with the auto‐mode (closed‐loop) function disabled. For those assigned to SAP therapy first, this sequence was reversed.

Sleep was assessed over 7 days, 12 weeks after the start of each intervention period using actigraphy (Philips Respironics, Murrysville, Pennsylvania). Data were analysed in 15‐second epochs with outcomes being: bedtime; wake time; sleep onset latency; total sleep time; sleep efficiency (%); wake after sleep onset (minutes); and number of awakenings.^8^

The Pittsburgh Sleep Quality Index (PSQI) questionnaire was completed at the end of each treatment period; each of its seven components have scores ranging from 0 (no difficulty) to 3 (severe difficulty); the Global score ranges from 0 to 21. Compared to polysomnography, a global PSQI score of >5 has been defined as “poor sleep” (sensitivity: 90%; specificity: 87%).^9^

“Night‐time” was defined as 12:00 am to 5:59 am. Glucose sensor outcomes were defined as the percentage of time in prespecified glucose ranges: 3.9 to 10.0 mmol/L; >10.0 mmol/L; and <3.9 mmol/L and <3.0 mmol/L.^10^

### 2.1 Statistical analysis

Analyses were performed on an intention‐to‐treat basis. Daily sensor glucose and actigraphy outcomes were aggregated across all nights across individual intervention periods. Correlation and scatter plots assessed relationships between glucose levels, actigraphy‐derived sleep traits and PSQI outcomes. Differences between study periods were assessed using mixed‐effects regression models. As this was a post hoc exploratory study, no a priori power calculation was performed, and no adjustments were made for multiple comparison. A *P* value < 0.05 was taken to indicate statistical significance. Sensor‐based glucose outcomes were calculated using GStat software, version 2.3 (University of Cambridge, Cambridge, UK). Daily and summative scores of actigraphy sleep measures were calculated using Actiware 6.0 software (Philips Respironics, Bend, Oregon). Statistical analyses were conducted using SPSS (IBM software, version 25).

## 3 RESULTS

Thirty‐seven participants were randomized (median [interquartile range] age 68 [63–70] years, 57% male, mean [standard deviation] baseline HbA1c 57.4 [9.6] mmol/mol or 7.4 [0.9]%). Time in target glucose range overnight was 10.7 percentage points higher (*P* < 0.001) in the HCL period compared with the SAP period (Table 1). Time spent with glucose at >10.0 mmol/L and mean glucose were lower using the HCL device versus the SAP (Table 1). Time spent in hypoglycaemia and glucose variability were comparable between the two treatment periods (*P* = 0.69).

**TABLE 1:** Between‐group differences in glucose and sleep outcomes at the end of intervention periods

Sleep outcomes	HCL (*n* = 34)	SAP (*n* = 36)	^a^ Paired mean difference	*P* value
Overnight (12:00–5:59 am) sensor glucose outcomes during objectively measured sleep				
Time spent at glucose level, %				
3.9 to 10.0 mmol/L	85.8 (11.3)	76.2 (15.3)	10.7 (95% CI 6.2, 15.1)	<0.001
>10.0 mmol/L	12.8 (11.5)	22.2 (15.6)	−10.4 (95% CI −14.9, −5.8)	<0.001
< 3.9 mmol/L	1.29 (0.00, 2.18)	0.10 (0.00, 2.58)		0.864
<3.0 mmol/L	0.00 (0.00, 0. 24)	0.00 (0.00, 0.00)		0.583
Mean glucose, mmol/L	7.5 (1.0)	8.3 (1.2)	−0.85 (95% CI −1.2, −0.51)	<0.001

Sleep outcomes	HCL (*n* = 35)	SAP (*n* = 36)	^a^ Paired mean difference	*P* value
Total sleep time, hours	7.6 (1.1)	7.5 (1.1)	0.13 (95% CI −0.25, 0.51)	0.491
Sleep onset latency, minutes	32.6 (20.5, 53.1)	32.5 (22.1, 68.5)		0.448
Sleep efficiency, %	81.5 (5.6)	80.0 (6.4)	1.9 (95% CI −0.016, 3.83)	0.052
Wake after sleep onset, minutes	37.3 (32.6, 60.8)	46.9 (30.9, 59.4)		0.480
Awakenings, number	50.1 (35.3, 65.7)	51.2 (41.7, 73.4)		0.257
Perceived sleep quality past month: PSQI score	5.3 (3.3)	5.2 (2.9)	−0.03 (95% CI −1.09, 1.02)	0.954

*Note*: Data are expressed as mean ± standard deviation or median (interquartile range).

Abbreviations: HCL; hybrid closed‐loop, PSQI, Pittsburgh Sleep Quality Index; SAP; sensor augmented pump.

^a^ Normally distributed data are presented as mean differences of values (HCL intervention minus SAP control phase). A positive difference indicates that the measurement was higher during the HCL period than during the SAP period.

The PSQI global and seven subscale scores were similar in the two groups. The proportion of participants with self‐reported poor sleep quality (PSQI score >5) was nonsignificantly higher during the SAP period compared to the HCL period (40.5 [24.8‐57.9]% vs. 29.7 [15.9‐47.0]%; *P* = 0.15).

Total sleep times during the HCL and SAP periods were similar and within the recommended 7 to 8 hours.^8^ Sleep efficiency was lower than the recommended >85% in both groups and was nonsignificantly higher in the HCL group (81.5 [5.6]% vs. 80.0 [6.4]%; *P* = 0.052). Sleep onset latency, wake after sleep onset and number of awakenings were all similar in the two groups.

Across the whole study period, longer time spent in hypoglycaemia (<3.9 mmol/L) and longer sleep onset latency were weakly correlated (*r* = 0.256, Supplemental Table S1 and Supplemental Figure S1). During the HCL period only, longer time spent within range 3.9 to 10.0 mmol/L was weakly correlated with longer sleep onset latency (*r* = 0.378), and longer time spent in hyperglycaemia >10.0 mmol/L was correlated with shorter sleep onset latency (*r* = −0.389; Supplemental Table S2, Supplemental Figures S2 and S3).

## 4 DISCUSSION

We observed improved glucose control with the CamAPS FX HCL device but no overall improvement in either subjectively or objectively defined sleep traits. In the whole cohort, longer time spent with hypoglycaemia was correlated with longer sleep onset latency. In the HCL period, longer time spent with normoglycaemia was correlated with longer sleep onset latency and longer time spent with hyperglycaemia correlated with shorter sleep onset latency.

Observational HCL studies using the Tandem Control IQ and the Medtronic 670G HCL systems have shown improvements in both glucose control and sleep.^6^, ^11^ However, potential study limitations include risk of confounding and assessment of subjective sleep traits only.

An RCT comparing glycaemic control achieved with the Medtronic 670G device versus an SAP in 30 older adults^12^ also assessed effects on sleep using actigraphy, sleep diary measures and PSQI score. Although the HCL device improved glucose control compared to the SAP, PSQI scores and actigraphy‐derived sleep quality did not improve and sleep quality recorded daily worsened, perhaps because of device alarms. There is increasing recognition of device‐induced sleep disruption and impaired well‐being, which can attenuate their recognized benefits.^5^ We found no change in sleep traits, possibly because any improvements in sleep achieved through improved normoglycaemia rates were offset by alarm‐related sleep disturbance. As with the aforementioned studies, we did not have systematic information about alarms, so we were not able to test this hypothesis.

While our study did not focus on sleep patterns of participants' partners or caretakers, the potential impact of the HCL device on this group warrants further attention. Qualitative studies suggest that diabetes technology use can sometimes be perceived negatively by partners and can adversely affect the couples' relationship,^13^, ^14^ partly as a result of device‐related sleep disturbance.^13^

Our observation of longer time spent in hypoglycaemia correlating with longer sleep onset latency is plausibly explained by anxiety and fear of hypoglycaemia induced by low sensor glucose values, which may have delayed sleep through sympathetic nervous system activation and stress hormone release.

During the HCL period, the association between longer time spent in hyperglycaemia and shorter sleep onset latency could also be explained by individuals perceiving a greater sense of safety through having a lower perceived risk of night‐time hypoglycaemia, and thus falling asleep more easily. Longer time spent in hyperglycaemia could potentially worsen other sleep traits through mechanisms including increased alarms, nocturia, painful neuropathy, and restless legs syndrome, but we observed no such relationships, possibly because the degree of hyperglycaemia was modest, and few participants had diabetes‐related complications.^7^

Our study's strengths include the randomized crossover study design which minimized risk of confounding. We assessed HCL efficacy and safety in older adults, a cohort usually excluded from diabetes technology studies. Objective and subjective sleep outcomes were assessed with internationally recommended standards of glucose sensor‐based outcomes.

Our study also had some limitations. It was a post hoc analysis of a previous study, hence it may have been underpowered to identify small treatment‐related differences in sleep traits. Glycaemic control at baseline was reasonable, with minimal differences in time below range between the groups. This and the relatively long time in range of the study participants may also limit generalizability. Other limitations were the lack of reliable data on alarms and fear of hypoglycaemia. Although actigraphy has relatively high agreement and low bias compared with the “gold standard” polysomnography for all sleep parameters, its agreement with sleep onset latency is reportedly less precise compared with polysomnography^15^, ^16^ and has not been validated for measuring sleep stages.^17^

In conclusion, improved glucose control with the CamAPS FX HCL system in older people was associated with no overall improvement in subjective or objectively defined sleep traits. The association between time spent in hyperglycaemia and sleep onset latency during the HCL period is of interest and warrants further investigation. There is also a clinical need to further understand the impact of devices on sleep health. Future studies should routinely report objective and subjective sleep outcomes, as well as examining factors likely to impact sleep including alarm burden, fear of hypoglycaemia, pre‐existing sleep patterns and the impact the HCL device may have on a partner's sleep quality. These strategies may potentially support choice of HCL systems in the future based on the individual's needs.

## CONFLICT OF INTEREST

Hood Thabit receives consulting fees and speaker honoraria from Eli Lilly, and reports having received research support from Dexcom Inc. Charlotte Boughton has received consulting fees from CamDiab. Julia K. Mader is a member on the advisory board of Abbott Diabetes Care, Boehringer Ingelheim, Becton‐Dickinson, Eli Lilly, Medtronic, NovoNordisk A/S, Prediktor A/S, Roche Diabetes Care and Sanofi‐Aventis, and has received speaker honoraria from Abbott Diabetes Care, Becton‐Dickinson, Dexcom, Eli Lilly, MSD, NovoNordisk A/S, Roche Diabetes Care, Sanofi, Servier and Ypsomed. Mark Evans is a clinical triallist with and/or has served on advisory boards and/or received speakers/writers fees from Medtronic, Dexcom, Abbott Diabetes Care, Roche, Astra Zeneca, Novo Nordisk, Eli Lilly, Zucara, Pila Pharma and Imcyse Pharma. Lalantha Leelarathna has received personal fees from Abbott Diabetes Care, Dexcom, Insulet, Medtronic, Novo Nordisk and Sanofi. Malgorzata E. Wilinska reports patents related to closed‐loop and being a consultant at CamDiab. Alice Gregory was CEO of Sleep Universal Ltd (2022) and an advisor for a project initially sponsored by Johnson's Baby. She is a consultant for Perrigo (2021+). She receives royalties for two books Nodding Off (Bloomsbury Sigma, 2018) and The Sleepy Pebble (Flying Eye, 2019). She has another contract with Lawrence King Publishers (publication due 2023). She is a regular contributor to BBC Focus magazine and has contributed to other outlets (such as The Conversation, The Guardian and Balance Magazine). She occasionally receives sample products related to sleep (e.g., blue light blocking glasses) and has given a paid talk to a business (Investec). She is a specialist subject editor at JCPP (sleep), for which she receives a small honorarium. She has contributed a paid article to Neurodiem. Roman Hovorka reports receiving speaker honoraria from Eli Lilly, Dexcom and Novo Nordisk, receiving licence and/or consultancy fees from B. Braun and Abbott Diabetes Care, patents related to closed‐loop, and being director at CamDiab. Martin K. Rutter, Womba Mubita, Jose Rubio and Catherine Fullwood declare no competing financial interests.

### PEER REVIEW

The peer review history for this article is available at https://publons.com/publon/10.1111/dom.14914.

## Supporting information


**Table S1.** Correlation for the whole group.
**Table S2.** Correlation for hybrid closed‐loop group only.
**Table S3.** Correlation for control group only.

**Figure S1.** Time with glucose <3.9 mmol/L vs. sleep onset latency for the whole group.
**Figure S2.** Time with glucose in range 3.9‐10.0 mmol/L vs. sleep onset latency for hybrid closed‐loop group only.
**Figure S3.** Time with glucose >10.0 mmol/L vs. sleep onset latency for hybrid closed‐loop group only.

## Data Availability

The data that support the findings of this study are available from the corresponding author upon reasonable request.
