# Supplementary material for: Impact of the CamAPS FX hybrid closed‐loop insulin delivery system on sleep traits in older adults with type 1 diabetes
Source: Diabetes Obes Metab. 2022 Nov 15;25(3):889–93. doi: 10.1111/dom.14914 (PMC9898088; doi:10.1111/dom.14914)
Supplement: Supplementary file 1 — Table S1. Correlation for the whole group. Table S2. Correlation for hybrid closed‐loop group only. Table S3. Correlation for control group only. [file DOM-25-889-s002.docx]

**Supplemental Table 1. Correlation for the whole group**

|  | Total sleep duration (mins) | Sleep onset latency (mins) | Sleep efficiency (%) | Wake after sleep onset (mins) | Number of awakenings |
| --- | --- | --- | --- | --- | --- |
| Time with glucose in range (%) | 0.072 | 0.056 | -0.123 | -0.073 | 0.100 |
| Time with glucose <3.9 mmol/l (%) | 0.056 | **0.256 *** | -0.009 | -0.176 | -0.167 |
| Time with glucose <3.0 mmol/l (%) | -0.123 | 0.074 | -0.046 | -0.102 | -0.106 |
| Time with glucose >10.0 mmol/l (%) | -0.073 | -0.133 | -0.060 | 0.070 | 0.164 |
| Time with glucose >16.7 mmol/l (%) | 0.100 | -0.110 | 0.173 | -0.110 | -0.070 |

* p <0.05

**Supplemental Table 2. Correlation for hybrid closed-loop group only**

|  | Total sleep duration (mins) | Sleep onset latency (mins) | Sleep efficiency (%) | Wake after sleep onset (mins) | Number of awakenings |
| --- | --- | --- | --- | --- | --- |
| Time with glucose in range (%) | -0.008 | **-0.378 *** | -0.068 | -0.235 | -0.245 |
| Time with glucose <3.9 mmol/l (%) | -0.007 | 0.250 | -0.008 | -0.183 | -0.273 |
| Time with glucose <3.0 mmol/l (%) | -0.103 | 0.258 | -0.220 | -0.067 | -0.287 |
| Time with glucose >10.0 mmol/l (%) | 0.012 | **-0.389 *** | 0.059 | 0.256 | 0.278 |
| Time with glucose >16.7 mmol/l (%) | 0.077 | -0.219 | 0.318 | -0.072 | -0.025 |

* p <0.05

**Supplemental Table 3. Correlation for control group only**

|  | Total sleep duration (mins) | Sleep onset latency (mins) | Sleep efficiency (%) | Wake after sleep onset (mins) | Number of awakenings |
| --- | --- | --- | --- | --- | --- |
| Time with glucose in range (%) | 0.103 | 0.047 | 0.065 | 0.101 | -0.050 |
| Time with glucose <3.9 mmol/l (%) | 0.140 | 0.270 | -0.035 | -0.146 | -0.019 |
| Time with glucose <3.0 mmol/l (%) | -0.227 | -0.072 | 0.127 | -0.152 | 0.143 |
| Time with glucose >10.0 mmol/l (%) | -0.108 | -0.137 | -0.070 | -0.077 | 0.065 |
| Time with glucose >16.7 mmol/l (%) | 0.148 | -0.105 | 0.152 | -0.155 | -0.119 |
