# Supplementary material for: Impact of the CamAPS FX hybrid closed‐loop insulin delivery system on sleep traits in older adults with type 1 diabetes
Source: Diabetes Obes Metab. 2022 Nov 15;25(3):889–93. doi: 10.1111/dom.14914 (PMC9898088; doi:10.1111/dom.14914)

**Supplemental Figures**

**S1. Time with glucose <3.9mmol/l vs. sleep onset latency for the whole group**


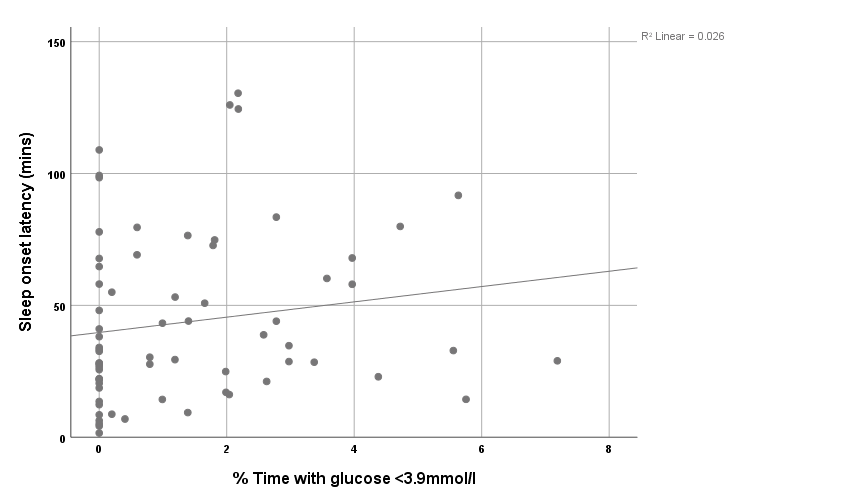


**S2. Time with glucose in range 3.9-10.0 mmol/l vs. sleep onset latency for hybrid closed-loop group only**
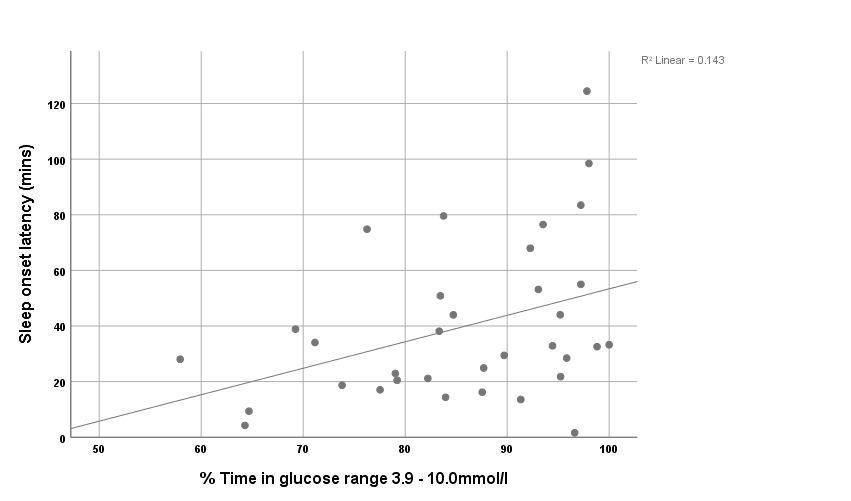


**S3. Time with glucose > 10.0 mmol/l vs. sleep onset latency for hybrid closed-loop group only**


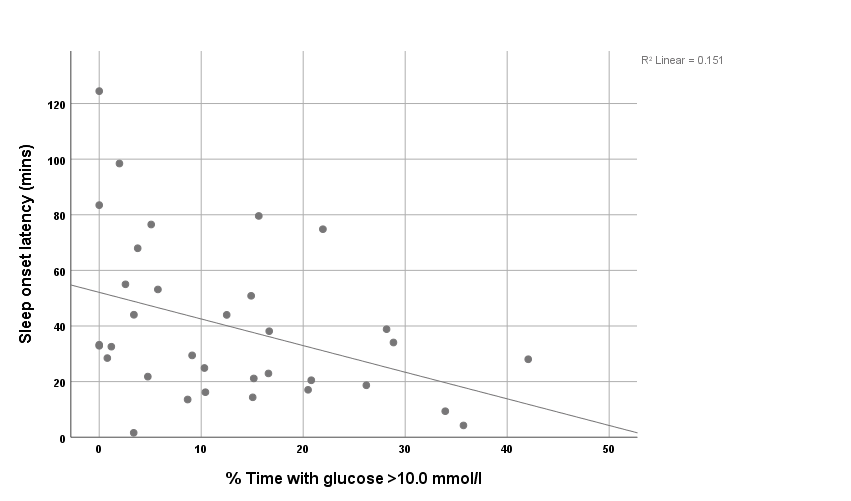

Supplement: Supplementary file 2 — Figure S1. Time with glucose <3.9 mmol/L vs. sleep onset latency for the whole group. Figure S2. Time with glucose in range 3.9‐10.0 mmol/L vs. sleep onset latency for hybrid closed‐loop group only. Figure S3. Time with glucose >10.0 mmol/L vs. sleep onset latency for hybrid closed‐loop group only. [file DOM-25-889-s001.docx]
